# Association between dietary oxidative balance score and constipation: evidence from National Health and Nutrition Examination Survey

**DOI:** 10.3389/fnut.2025.1509687

**Published:** 2025-02-27

**Authors:** Zhangpeng Shi, Dandan Wang, Jiacheng Yu, Mengting Zhou, Jiahao Du, Huanlong Qin, Huiyuan Zhu

**Affiliations:** ^1^Department of Gastrointestinal Surgery, Shanghai Tenth People’s Hospital, Tongji University School of Medicine, Shanghai, China; ^2^Research Institute of Intestinal Diseases, Tongji University School of Medicine, Shanghai, China; ^3^Department of Gastrointestinal Surgery, Shanghai Tenth People’s Hospital, School of Clinical Medicine of Nanjing Medical University, Shanghai, China; ^4^Department of Pathology, Shanghai Tenth People’s Hospital, Tongji University School of Medicine, Shanghai, China

**Keywords:** constipation, dietary oxidative balance scores, dietary factors, National Health and Nutrition Examination Survey, cross-sectional analysis

## Abstract

**Background:**

Numerous researches have revealed a correlation between dietary factors and the development of constipation. This study aimed to investigate the association between dietary oxidative balance score (DOBS) and constipation.

**Materials and methods:**

A cross-sectional study was conducted by us based on the National Health and Nutrition Examination Survey (NHANES) from 2005 to 2010 including 31,034 individuals who completed a constipation questionnaire. The DOBS was calculated based on 16 dietary factors, containing 14 antioxidants and two prooxidants. Multiple logistic regression and restricted cubic spline (RCS) analyses were employed to examine the correlation between DOBS and constipation. Meanwhile, propensity score matching (PSM) was chosen to eliminate the effect of confounding variables.

**Results:**

A total of 11,019 participants were identified as constipation. After adjusting for potential confounding variables, multiple logistic regression analysis indicated that decreasing DOBS (OR = 0.977, 95% CI: 0.966–0.987, *p* < 0.001) was apparently associated with increased risk of constipation incidence. Notably, the occurrence of constipation increased with reduced level of DOBS, as compared to Q1 (Q2, OR = 0.820, 95% CI, 0.682–0.988, *p* = 0.037; Q3, OR = 0.797, 95% CI, 0.653–0.973, *p* = 0.026; Q4, OR = 0.648, 95% CI, 0.528–0.797, *p* < 0.001).

**Conclusion:**

Low levels of DOBS were positively associated with the risk of constipation development, demonstrating that DOBS could be employed as a dietary indicator of constipation prevention.

## Introduction

Constipation, a widespread gastrointestinal disorder, characterized by incomplete evacuation, infrequent bowel movements, and a sense of anorectal obstruction, seriously compromises patients’ quality of life and increases healthcare costs. The global prevalence of constipation is approximately 15%, with higher rates observed among old adults, females, and individuals with lower socioeconomic status (1, 2).

The pathogenesis of constipation is complex. Current mainstream recognition indicates that colonic sensorimotor disorders, pelvic floor dysfunction, dietary factors, disturbances of intestinal flora, and use of some medications are closely related to constipation (2). Treatment options available for constipation, including dietary prevention, medications, mechanical stimulation and microecological regulators, only provide temporary relief and lead to some side effects (3–6). Recently, dietary antioxidants have shown a great promise in the treatment of constipation (4, 7, 8). However, if the impacts of oxidative stress in dietary prooxidants such as fatty foods, and high iron intake are ignored during diet, it is likely that the overall effectiveness of the diet in preventing constipation will be poor (9–11). Consequently, seeking a dietary oxidative balance predictor is of significance to understand and manage constipation.

It is widely known that dietary factors play a vital role in the prevention and treatment of constipation. Numerous researches uncovered the inverse associations between the occurrence of constipation with the intakes of antioxidants vitamin B1, vitamin E, protein, copper, selenium, and niacin (12–17). However, dietary factors are complicated and difficult to control, and it is meaningful to consider dietary balance systematically and comprehensively. Herein, we integrated the following dietary factors including dietary fiber, β-carotene, calcium, magnesium, zinc, copper, selenium, niacin, total folate, vitamin B2, vitamin B6, vitamin B12, vitamin C, vitamin E, total fat, and iron for scoring, and defined as dietary oxidative balance score (DOBS), acting as a comprehensive indicator and reflecting the overall exposure balance of dietary antioxidants and prooxidants. The purpose of this study was to evaluate the association between DOBS and constipation via a cross-sectional analysis from National Health and Nutrition Examination Survey.

## Materials and methods

### Study population

**Figure 1:**
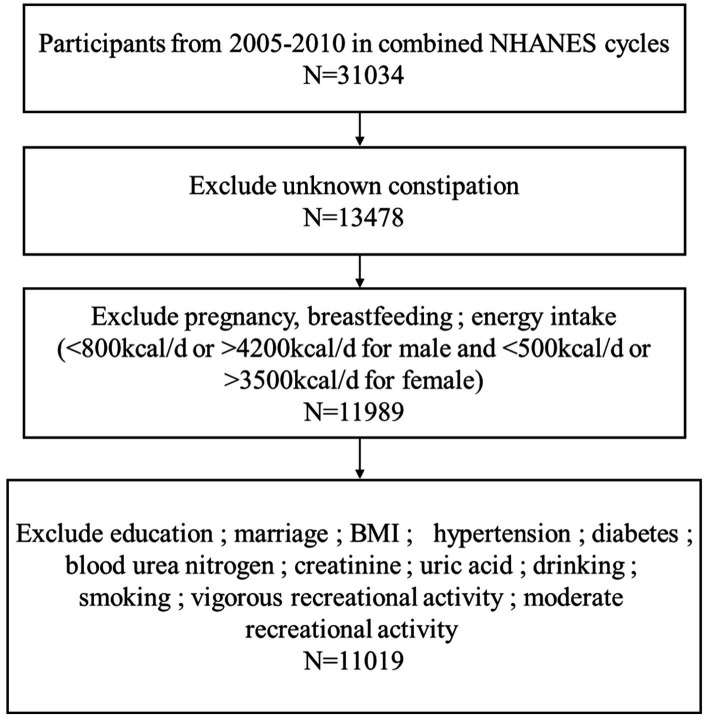
Schematic flow diagram of inclusion and exclusion criteria in this study cohort.

The study carried out a cross-sectional analysis of publicly available NHANES database from 2005 to 2010, totally including 31,034 participants surveyed. The exclusion criteria are as follows: (1) participants who did not know if they suffered from constipation, *n* = 17,556; (2) individuals were excluded if they were pregnant, breastfeeding, or their energy intakes were incredible (<500 kcal/d or > 3,500 kcal/d for female; <800 kcal/d or > 4,200 kcal/d for male), *n* = 1,489; (3) participants who had missing data on education, marital status, body mass index (BMI), hypertension, diabetes mellitus, blood urea nitrogen, creatinine, uric acid, Gamma-glutamyl transferase (GGT), alcohol consumption, mild and strenuous recreational activities, and cigarette smoking, *n* = 970. According to the exclusion criteria, 11,019 participants were finally included in the present study (Figure 1).

### Dietary oxidative balance scores

In short, DOBS is the sum of 16 dietary factor scores. We calculated each individual’s DOBS using the method reported in the previous literature. Based on this method, 16 components of DOBS were divided into two groups: (1) dietary antioxidants (dietary fiber, carotene, riboflavin, niacin, vitamin B6, total folate, vitamin B12, vitamin C, vitamin E, calcium, magnesium, zinc, copper, and selenium), and (2) dietary prooxidants (iron and total fat). Furthermore, all dietary components were categorized into three tiers according to their distribution in this study population. The above antioxidants were allocated points from 0 to 2 from tertile 1 to tertile 3, respectively, while the point assignment for prooxidants was reverse, with 2 points for the lowest tertile and 0 points for the highest tertile. Finally, the overall DOBS was calculated by summing the scores for the 16 dietary components, ranging from 0 to 32 points.

### Assessment of constipation

The NHANES employs defecation habits to investigate constipation in participants between 2005 and 2010 through completing the Bowel Health Questionnaire conducted in Mobile Examination Centers. Participants with fewer than 2 defecation per week are defined as constipated, those with 3–21 movements per week are considered normal, and more than 21 defecation frequency per week indicates diarrhea. Moreover, individuals are classified as constipated if they self-report type 1 (separate hard lumps) or type 2 (sausage-like with lumps) stools based on the Bristol Stool Classification.

### Covariates

Besides, we included the following factors in the study: gender (male, female), age (≤49 years, >49 years), race (Non-Hispanic White, other race), education (≤High school, >High school), marital status, body mass index (BMI, ≤28, >28), hypertension, diabetes, blood urea nitrogen, creatinine, uric acid, GGT, alcohol consumption, smoking, vigorous recreational activity, moderate recreational activity and DOBS.

### Statistical analysis

In this study, mean with standard deviation (SD) represented continuous variables, while the median (upper and lower quartiles) was used to denote categorical variables. Furthermore, a weighted *t*-test was applied to analyze continuous variables and a chi-square test to compare categorical variables. Univariate and multifactorial logistic regression analyses were performed to explore the prevalence of constipation and analyze the differences. Additionally, we used a restricted cubic spline function to describe the dose–response relationship between the continuous variables DOBS with constipation. Moreover, multiple logistic regression models were utilized to evaluate the odds ratio (OR) and 95% confidence interval (CI). In the basic model, we adjusted the model based on variables gender, age, race, education and married status, and the core model further added hypertension, diabetes, blood urea nitrogen and uric acid to the base model. Considering the overall quality of life of participants, GGT, smoking, alcohol consumption, vigorous recreational activity and moderate recreational activity were added to the core model to produce an extended model. Statistical analyses were performed with SPSS (version 24.0) software, and graphs were created with R (version 4.1.3) and Graph Pad program (version 9.0.0) software. A *p*-value less than 0.05 was considered as statistically significant.

## Results

### Participants characteristics

Based on the process criteria in Figure 1, a total of 11,019 participants from the NHANES between 2005 and 2010 were enrolled in our study. Table 1 presented the baseline clinical characteristics of the participants, of whom 891 (8.1%) self-reported as patients suffered from constipation and 10,128 (91.9%) had no constipation manifestation as controls. Then a chi-square analysis was used to estimate the clinical characteristics of all individuals. It was found that gender (*p* < 0.001), age (*p* = 0.003), race (*p* < 0.001), education level (*p* < 0.001), marital status (*p* < 0.001), BMI (*p* = 0.002), hypertension (*p* = 0.018), blood urea nitrogen (*p* = 0.035), creatinine (*p* < 0.001), uric acid (*p* < 0.001), GGT (*p* = 0.007), alcohol use (*p* < 0.001), smoking (*p* < 0.001), vigorous recreational activity (*p* < 0.001), moderate recreational activities (*p* = 0.002) and DOBS (*p* < 0.001) were significantly different between patients with constipation and controls. Specifically, patients with constipation are more likely to be female, younger, drinkers, Non-Hispanic White individuals, poorly educated, unmarried, no history of hypertension and smoking, lower density recreational activity, lower level of BMI, blood urea nitrogen, creatinine, uric acid, GGT, and DOBS.

**Table 1:** Baseline characteristics of NHANES participants between 2005 and 2010 (*n* = 11,019).^a^

Characteristic	All	None constipation	Constipation	*p* value
	Patients	No. 91.9 (%)	No. 8.1 (%)	
	*N* = 11,019	*N* = 10,128	*N* = 891	
Gender				<0.001
Male	5,511 (50.0)	5,233 (51.7)	278 (31.2)	
Female	5,508 (50.0)	4,895 (48.3)	613 (68.8)	
Age				0.003
Age ≤ 49	5,564 (50.5)	5,072 (50.1)	492 (55.2)	
Age > 49	5,455 (49.5)	5,056 (49.9)	399 (44.8)	
Race				<0.001
Non-Hispanic White	5,631 (51.1)	5,237 (51.7)	394 (44.2)	
Other race	5,388 (48.9)	4,891 (48.3)	497 (55.8)	
Education				<0.001
≤High school	5,537 (50.2)	5,007 (49.4)	530 (59.5)	
>High school	5,482 (49.8)	5,121 (50.6)	361 (40.5)	
Marital status				<0.001
Married	5,959 (54.1)	5,533 (54.6)	426 (47.8)	
Unmarried	5,060 (45.9)	4,595 (45.4)	465 (52.2)	
BMI				0.002
≤28	5,573 (50.6)	5,081 (50.2)	492 (55.2)	
>28	5,446 (49.4)	5,047 (49.8)	399 (44.8)	
Hypertension				0.018
Yes	3,737 (33.9)	3,464 (34.2)	270 (30.3)	
No	7,282 (66.1)	6,661 (65.8)	621 (69.7)	
Diabetes				0.715
Yes	1,233 (11.2)	1,130 (11.2)	103 (11.6)	
No	9,786 (88.8)	8,998 (88.8)	788 (88.4)	
Blood urea nitrogen				0.035
≤12	5,674 (51.5)	5,185 (51.2)	489 (54.9)	
>12	5,345 (48.5)	4,943 (48.8)	402 (45.1)	
Creatinine				<0.001
≤0.87	5,516 (50.1)	4,990 (49.3)	526 (59.0)	
>0.87	5,503 (49.9)	5,138 (50.7)	365 (41.0)	
Uric acid				<0.001
≤5.4	5,785 (52.5)	5,223 (51.6)	562 (63.1)	
>5.4	5,234 (47.5)	4,905 (48.4)	329 (36.9)	
GGT				0.007
≤21	5,878 (53.3)	5,364 (53.0)	514 (57.7)	
>21	5,141 (46.7)	4,764 (47.0)	377 (42.3)	
Alcohol consumption				<0.001
Yes	7,986 (72.5)	7,429 (73.4)	557 (62.5)	
No	3,033 (27.5)	2,699 (26.6)	334 (37.5)	
Smoking				<0.001
Yes	5,178 (47.0)	4,822 (47.6)	356 (40.0)	
No	5,841 (53.0)	5,306 (52.4)	535 (60.0)	
Vigorous recreational activity				<0.001
Yes	2,616 (23.7)	2,444 (24.1)	172 (19.3)	
No	8,403 (76.3)	7,684 (75.9)	719 (80.7)	
Moderate recreational activity				0.002
Yes	4,855 (44.1)	4,506 (44.5)	349 (39.2)	
No	6,164 (55.9)	5,622 (55.5)	542 (60.8)	
DOBS (median [IQR])	16 [10,21]	16 [11,22]	14 [9,19]	<0.001

BMI, body mass index; GGT, gamma-glutamyl transferase; DOBS, dietary oxidative balance score.

a For categorical variables, *p* values were analyzed by chi-square tests. For continuous variables, the *t*-test for slope was utilized in generalized linear models.

### DOBS and constipation

**Figure 2:**
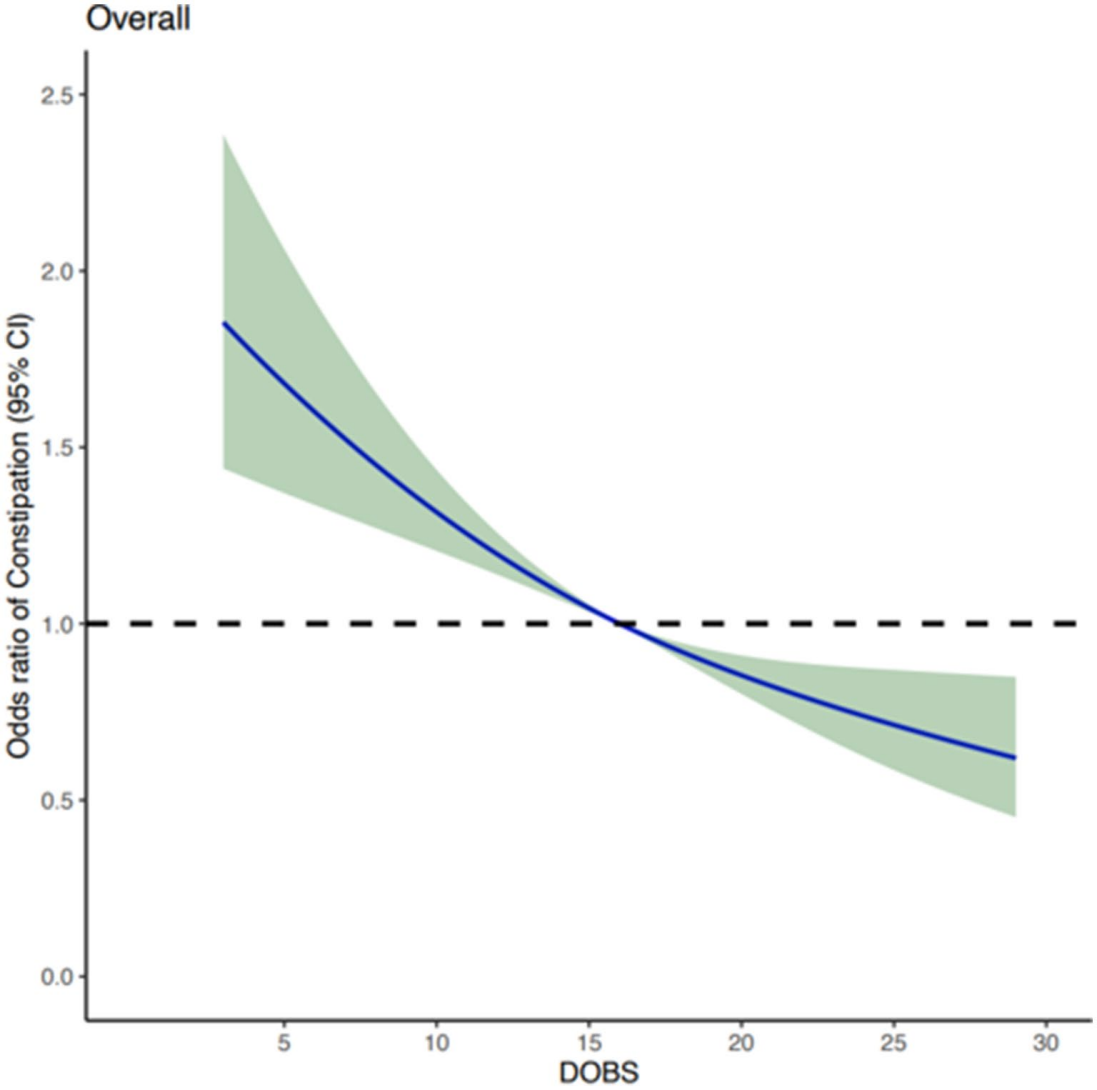
Dose–response curve presenting the association DOBS and the risk of constipation.

**Figure 3:**
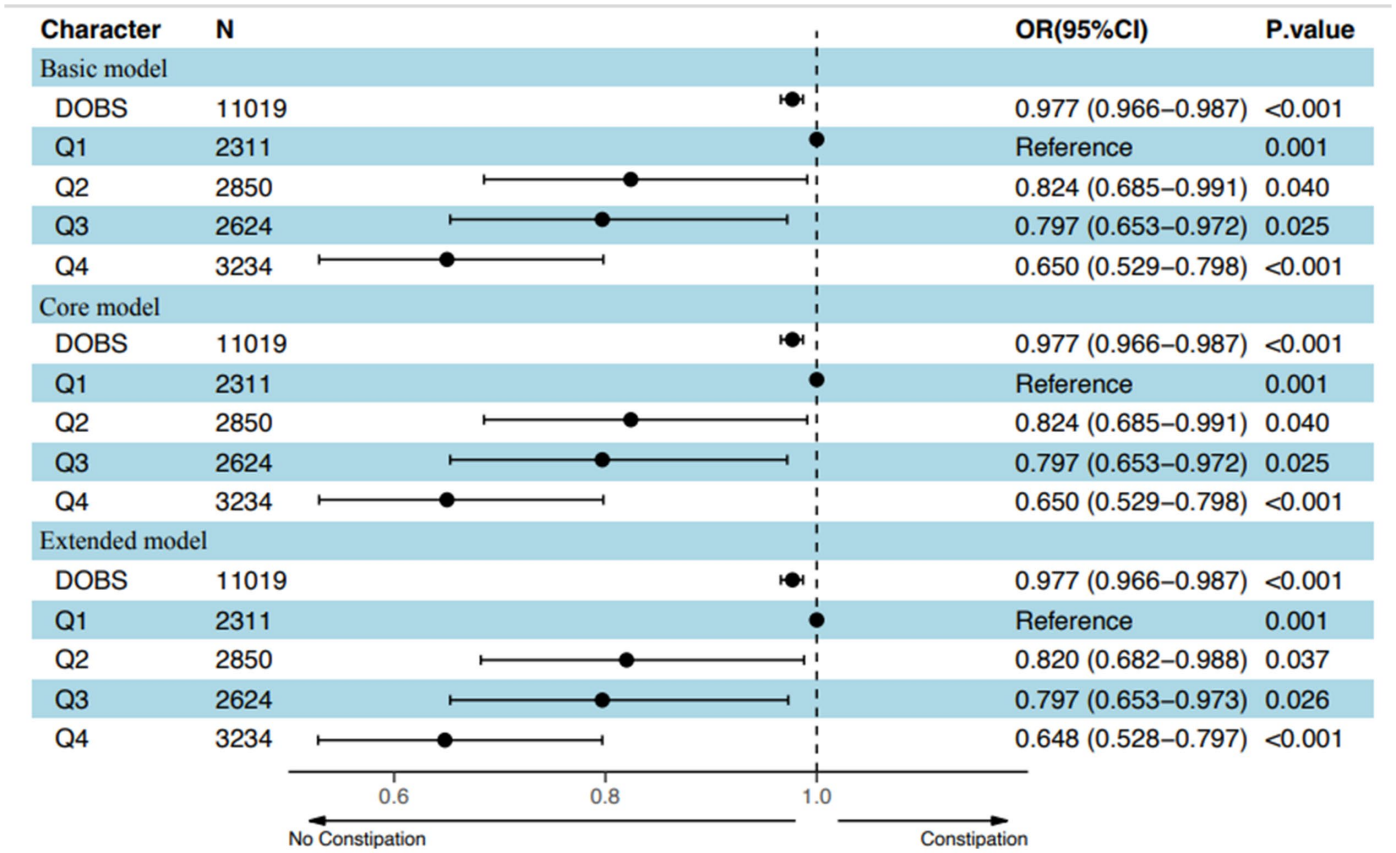
Adjusted odds of constipation decreased with increasing values of DOBS based on an overall model adjusted for potential confounders in the US population. Q stood for Quartile.

After adjusting for potential confounding covariates, dose curve analysis result demonstrated that the risk of developing constipation increased with elevated values of DOBS, indicating an apparent negative correlation between DOBS and constipation (Figure 2). Moreover, multivariate and univariate logistic regression analyses were performed to further identify the risk factors associated with the relationship between DOBS and constipation. Our findings illustrated that in three models including the basic model, the core model, and the extended model, constipation developed with the decreasing DOBS (All *p* < 0.001), as shown in Figure 3. Particularly, in the extended model, the adjusted OR for constipation varied with DOBS (Q2 vs. Q1: OR = 0.820, 95% CI, 0.682–0.988, *p* = 0.037; Q3 vs. Q1: OR = 0.797, 95% CI, 0.653–0.973, *p* = 0.026; Q4 vs. Q1: OR = 0.648, 95% CI, 0.528–0.797, *p* < 0.001), suggesting the significant negative association between them.

**Figure 4:**
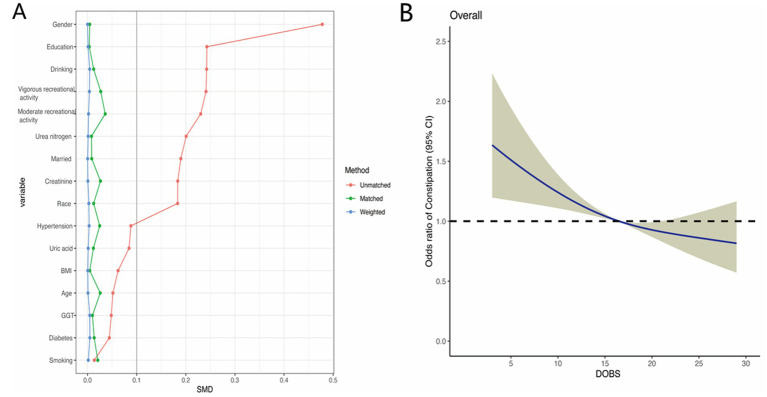
**(A)** Propensity score matching analyses performed on the standardized mean difference results for the different variables; **(B)** Dose–response analysis between DOBS and presence of constipation after propensity score matching.

**Figure 5:**
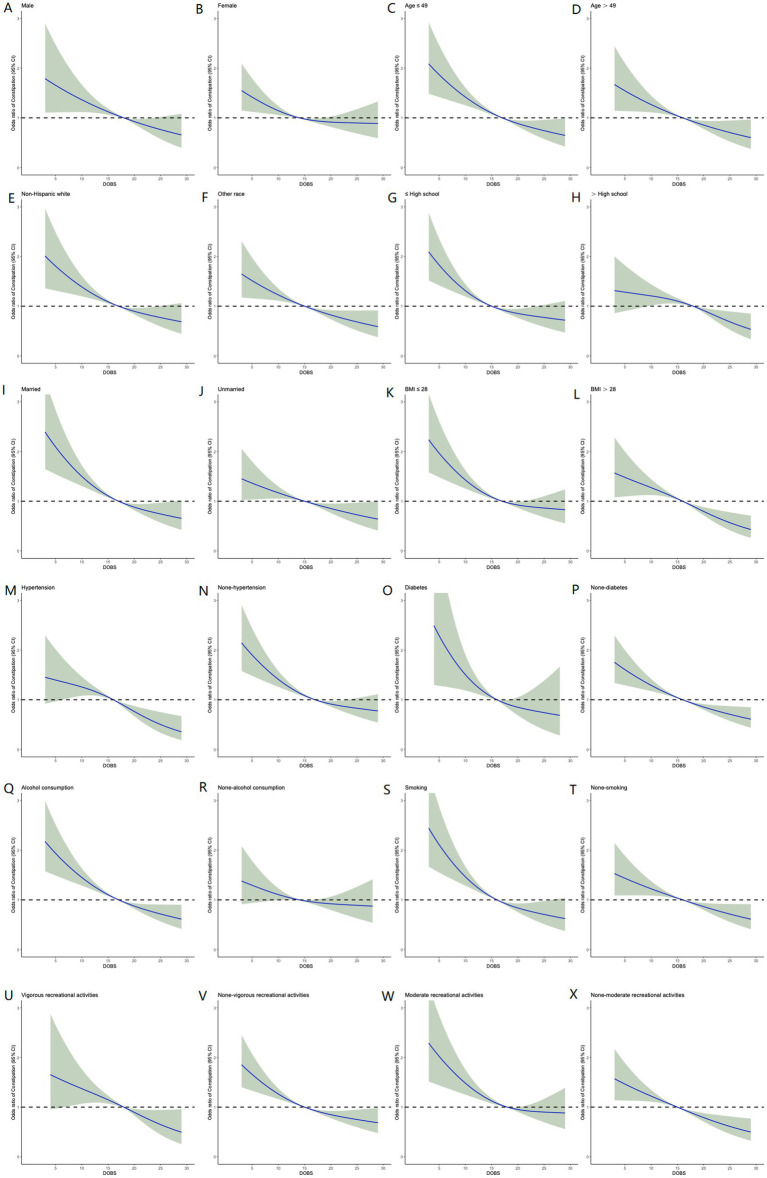
Relative risk of constipation based on DOBS levels in male **(A)**, female **(B)**, age ≤ 49 **(C)**, age > 49 **(D)**, Non-Hispanic White **(E)**, other race **(F)**, ≤high school **(G)**, >high school **(H)**, married **(I)**, unmarried **(J)**, BMI ≤ 28 **(K)**, BMI > 28 **(L)**, hypertension **(M)**, none-hypertension **(N)**, diabetes **(O)**, non-diabetes **(P)**, alcohol consumption **(Q)**, non-alcohol consumption **(R)**, smoking **(S)**, non-smoking **(T)**, vigorous recreational activity **(U)**, none-vigorous recreational activity **(V)**, moderate recreational activity **(W)**, and none-moderate recreational activity **(X)**.

To reduce the selectivity bias introduced by covariates, propensity score matching analyses were performed as displayed in Figure 4A. It was found that a more significant invert relationship between DOBS and constipation occurred as compared to Figure 2, after adjusting for nine confounding factors such as gender, education, etc. (Figure 4B). Besides, the dose–response analysis curves of restricted cubic spline function from the subgroup analysis further revealed that the risk of the incidence of constipation was negatively correlated with the level of DOBS (Figure 5).

## Discussion

In the present study, we investigated the association between DOBS and constipation involving 11,019 participants of American adults recorded in NHANES database through a cross-sectional analysis. All data and study population satisfy standard quality checks for authenticity and authority. Baseline characteristics data suggested that patients with constipation presented a lower DOBS as compared to those without constipation. After adjusting for potential confounding factors, it was found that reduced DOBS levels were associated with the increased risk of constipation, demonstrating that patients with the lower quartiles were more prone to having constipation. Moreover, dose-response curve between DOBS levels and constipation were established to further visualize the negative correlation between DOBS and constipation. Finally, subgroup analyses based on age, gender, race, education level, marital status, BMI, hypertension, diabetes, alcohol, smoking, and recreation activities, further verified the robustness of above results.

In recent years, the incidence of constipation has remained high globally, causing great pain, psychological and economic burdens to patients. Numerous studies have shown that constipation is closely linked to dietary factors and intestinal microbiota imbalance. Du et al. uncovered constipation symptoms can be alleviated by dietary trace element copper involving its abilities for growing intestinal motility and softening stools, showing an inverse relationship between dietary copper consumption and chronic constipation (13). Wang et al. found that, selenium, also as an essential trace element for the human body, was negatively correlated with the incidence on chronic constipation rather than chronic diarrhea (14). Besides, selenium or selenoprotein can decrease the level of intestinal oxidative stress by scavenging reactive oxygen species, remodeling the intestinal microbiota, and immunomodulation (18, 19). In addition to the dietary antioxidants mentioned above, vitamins including vitamin B1, vitamin E, and niacin also exhibited a negative association with the development of constipation (15–17). These antioxidant vitamins were able to alleviate the inflammatory response, repair the intestinal barrier as well as regulate the microbiota, thus favoring intestinal health. Based on the above evidence, we speculated that dietary factors can influence constipation, to a great extent by modulating the intestinal flora and redox homeostasis. Consequently, microecological modifiers (probiotic, prebiotic, postbiotic, synbiotic) may show good therapeutic promise in constipation. For instance, Ma et al. conducted a randomized, double-blind, placebo-controlled study involving 163 constipation patients. The study indicated that, Lactiplantibacillus plantarum P9, a representative probiotic, enabling regulating patients’ gut microbiota, bacteriophage composition, and microbial metabolites through various mechanisms and metabolic pathways, significantly improved the weekly mean frequency of complete spontaneous bowel movements and their quality of life (20). Additionally, fecal microbiota transplantation (FMT), referring to the gut microbiotas from healthy donors to patients to treat various diseases, may favor curing constipation. Our team’s research found that, compared to pre-FMT, post-FMT patients performed the increased α-diversity of intestinal flora and lowered serum IL-8, thereby improving functional constipation (21).

Diet factors, containing diet antioxidants and prooxidants, were complicated and difficult to control. To better reflect individual dietary oxidative homeostasis status, we included a total of 16 dietary factors and scored them according to previously reported methods from NHANES database. Particularly, in the extended model, the adjusted OR for constipation varied with DOBS (Q2 vs. Q1: OR = 0.820, 95% CI, 0.682–0.988, *p* = 0.037; Q3 vs. Q1: OR = 0.797, 95% CI, 0.653–0.973, *p* = 0.026; Q4 vs. Q1: OR = 0.648, 95% CI, 0.528–0.797, *p* < 0.001), indicating that people with lower DOBS were prone to have constipation. Of course, there are some constraints to this study. Firstly, the data in this work are from cross-sectional studies unable to determine a causal relationship between the occurrence of constipation and DOBS. Secondly, the average of two 24-h recalls to assess dietary components is likely to be biased. Thirdly, certain covariables were not encompassed in the analyses due to the absence of data for all the volunteers, such as use of medication enabling affecting constipation. Besides, reliance on self-reported dietary data in the study may create potential bias. Moreover, our findings are based on NHANES data from the US, limiting extrapolation to other populations. Finally, defining constipation based on questionnaires might not capture all clinical aspects of the condition. Further prospective studies are necessary to verify the association between constipation and DOBS.

## Conclusion

In summary, our study found a significant inverse association between dietary oxidative balance score (DOBS) and constipation incidence among United States adults, indicating that DOBS is able to function as a predictor for preventing and managing constipation.

## Data Availability

The original contributions presented in the study are included in the article/supplementary material, further inquiries can be directed to the corresponding authors.
